# Welfare Impacts of Pindone Poisoning in Rabbits (*Oryctolagus cuniculus*)

**DOI:** 10.3390/ani6030019

**Published:** 2016-02-26

**Authors:** Penny Fisher, Samantha Brown, Jane Arrow

**Affiliations:** Landcare Research, P.O. Box 69040, Lincoln 7640, New Zealand; browns@landcareresearch.co.nz (S.B.); arrowj@landcareresearch.co.nz (J.A.)

**Keywords:** animal welfare, anticoagulant, rabbit, pindone, poisoning

## Abstract

**Simple Summary:**

The nature and duration of the effects of pindone poisoning in rabbits were evaluated through observational monitoring of affected animals and necropsy. Using the resulting data in a formal assessment framework, the welfare impacts of pindone poisoning were ranked as relatively higher than other vertebrate toxic agents currently used for rabbit control.

**Abstract:**

Control methods used to manage unwanted impacts of the European rabbit in Australia and New Zealand include the use of toxic bait containing the anticoagulant pindone. Towards increased certainty in evaluating the animal welfare impacts of pindone poisoning in rabbits, we recorded behavioral and post-mortem data from rabbits which ingested lethal quantities of pindone bait in a laboratory trial. Pindone poisoning in rabbits resulted in welfare compromise, primarily through functional impairments related to internal haemorrhage over a maximum duration of 7 days. Applying this data to a formal assessment framework for ranking animal welfare impacts indicated that pindone had relatively high severity and also duration of welfare impacts in comparison to other rabbit control methods.

## 1. Introduction

Those tasked with managing impacts or populations of unwanted (“pest”) wild animals often have a variety of lethal and non-lethal control methods available to them. While cost-efficacy and human safety have been traditional drivers of the development, selection and application of control methods, animal welfare outcomes of pest animal management is an increasingly important criterion (e.g., [1,2]). In turn, this raises the need for some means of objectively evaluating welfare of a range of control methods as applied to a range of species [3].

One such example is the management of European rabbits (*Oryctolagus cuniculus*), a species introduced to New Zealand and Australia following British colonisation. In both countries, establishment and expansion of rabbit populations into favourable habitats was accompanied by unwanted effects on biological conservation and agricultural production values. Rabbits can compete with grazing livestock and some native species for food, cause soil erosion, modify native vegetation and influence dynamics of predator-prey interactions [4]. Management actions to mitigate such effects have utilized non-lethal methods such as exclusion fencing or chemical deterrents, but typically aim to cause significant, rapid reductions in rabbit populations through lethal methods such as warren destruction, shooting, trapping, disease introduction (myxomatosis and Rabbit Haemorrhagic Disease Virus) and use of vertebrate toxic agents (VTAs) either applied as toxic bait or as burrow fumigants [5,6].

In the context of using VTAs to control unwanted animals, early approaches to evaluating the humaneness of poisoning [7,8] identified central tenets based on the nature, severity and duration of harm caused by a particular toxic substance, qualified by the number of animals likely to be affected by operational practice of toxic bait application. This was further developed in a protocol [9] subsequently used to assess effects of particular VTAs in brushtail possums (*Trichosurus vulpecula*), which are considered a significant pest in New Zealand [10,11]. More recently, an evaluation framework [12] was designed to enable comparisons of welfare impacts across lethal and non-lethal control methods (not just VTAs) and across a range of unwanted animal species, while taking account of the Five Domains model of animal welfare [13]. The framework involves interpretation of available research data and technical information within a matrix (severity of effects *vs.* duration) for each method/animal species combination to produces scores, which can then be ranked in terms of relative welfare impacts. This framework [12], with some modifications, was applied to assessing lethal methods of pest animal management used in New Zealand [14]. In compiling that evaluation, variable amounts of information were available in relation to the effects on particular animals of the respective lethal control methods assessed, and this affected the certainty with which scores could be allocated to domain impacts so that expert opinion, assumption and/or extrapolation were used to derive a welfare score. A number of particular information gaps were identified which, if addressed, would improve the robustness of relative assessments of welfare impacts.

One such information gap concerned the use of the VTA pindone (2-pivaloyl-1,3-indandione) for rabbit control. Pindone is a first-generation indandione anticoagulant and its main toxic activity is to inhibit Vitamin K metabolism in the liver to the point where depletion of circulating active blood clotting factors results in failure of coagulation and fatal haemorrhage [15]. Paparella [16] described the use of anticoagulants in rodent management as a welfare paradox, with regard to the large numbers of animals (rodents) affected by the “markedly inhumane” process of anticoagulant poisoning. This concern is also applicable to the use of pindone in rabbit management [17]. We had the opportunity to address this information gap during a laboratory trial that aimed to measure residual concentrations of pindone in the tissues of poisoned rabbits. While an evaluation of welfare impacts was not the *raison d'être* of the study [18] within the study design, it was consistent with application of a knowledge-based ethic (e.g., [19]) to record additional behavioral and post-mortem data towards reducing uncertainty in scoring welfare impacts of pindone poisoning within a formal assessment framework.

## 2. Experimental Section

The study was conducted in 2011 under approval from the Landcare Research Animal Ethics Committee (Project No. 10/11/01). Laboratory housing and acclimation conditions for rabbits are described fully by Fisher *et al*. [18]. In brief, New Zealand White rabbits at least ten weeks old and of both sexes were housed individually in metal cages on a maintenance diet of feed pellets, supplemented by carrots, apples and lucerne hay with water freely available. Rabbits were offered cereal pellet bait containing 0.25 g/kg pindone in three trials (Table 1) where bait treatments were placed in their cages each morning and the following morning any remaining bait was weighed to determine the amount consumed. At the end of pindone bait presentation, all rabbits were returned to full maintenance diet for a 21 d monitoring period.

These treatments were intended to simulate low and high lethal intakes that could occur in operational application of bait for rabbit control. In Australia and New Zealand, carrot, oat or cereal pellet baits are used in bait station or broadcast applications, where rabbits typically have access to bait over a number of days. As for other ‘first generation’ anticoagulants, pindone is most effective when ingested in multiple, consecutive exposure over a few days, but a relatively much higher single exposure can also be lethal [20].

During these trials we sought to simulate movement and conspecific interactions typical of free-ranging rabbits, as potentially important influences on the effects of anticoagulant ingestion. Rabbits were acclimated for a minimum of two weeks to free exercise in same-sex groups in a 3 m^2^ floor enclosure for a minimum of one hour per day. Voluntary ingestion of potentially lethal quantities of pindone bait by rabbits was required to meet the main objective of the trials, which was to quantify residual pindone concentrations in rabbit tissue [18]. We sought to minimise welfare impacts through alternative endpoints that triggered killing of rabbits by intracardiac injection of sodium pentobarbitone. 

These endpoints were based on described effects of anticoagulant poisoning in rats and possums [21]. They were (i) loss of more than 25% of bodyweight from the start of a trial (ii) severe or extensive haemorrhage evident on external examination e.g., uncoagulated bleeding from an injury or orifice, bruised /swollen appearance of an area or limb, worsening lameness and (iii) loss of righting reflex, prostration and/or greatly reduced response to touch, noise or pinch stimuli. Any one or a combination of these states was presumed to reliably indicate eventual mortality through pindone poisoning. Qualitative, descriptive data recorded by technically experienced observers was supplemented by a scoring system (Table 2) based on the expected effects specific to pindone poisoning in rabbitsof anticoagulant poisoning described in other mammals [10]. 

Behavioural observations of each rabbit were recorded against time from the first potential intake of pindone bait, and recorded during the free exercise periods and at least twice-daily observations (morning and afternoon) of rabbits in their individual cages. Frequency of observations was increased to three-hour intervals where individual rabbits appeared to be approaching any endpoint *i.e.*, advanced progression of pindone poisoning. This enabled relatively precise estimates of times to first visible signs of poisoning, and description of the progression of poisoning to mortality. Statistical comparison of times to first signs of poisoning, and times to death was through one-way ANOVA and Tukey multiple comparisons of means in the program R (*version* 3.1.2, R Core Team 2014).

After pindone bait was offered monitoring of rabbits continued for 21 days. Any rabbits alive after that period were assumed to have been sublethally exposed or not exposed to pindone at all (as measured by bait intake) and were killed by lethal intracardiac injection. Post-mortem, all rabbits were stored chilled (−4 °C) for 3–24 h prior to necropsy and sampling of liver, muscle and fat tissue to test for residual concentrations of pindone (as described in [18]). At necropsy, weight of whole-body, liver and stomach contents were recorded along with general observations of gross pathology, especially the presence, extent and location of internal haemorrhage. 

During July–August 2011, intact rabbit carcasses were collected after two pindone-pellet baiting operations in the South Island of New Zealand, one in a pine plantation at the northern end of Lake Tekapo and the other at various locations around Wanaka (Clutha River, Stephensons Island, Poison Creek). Whole carcasses were stored frozen for up to two weeks, then defrosted and necropsied as for the laboratory rabbits.3. 

## 3. Results and Discussion

### 3.1. Laboratory Trials

Table 3 describes the numbers of rabbits that ingested different lethal amounts of pindone bait in the three laboratory trials. Overall the first evident signs of poisoning (to observers) occurred 6–12 days after bait was first eaten and typically included anaemia (pale ears or nose), reduced food intake, ungroomed appearance, lethargy/reluctance to move or outright lameness. One or a combination of these signs were observed on average 8.5 days after first bait ingestion with no significant differences between rabbits in the three trials (F_2,24_ = 1.1854, *p* = 0.32). “Illness” behaviours during the progression of poisoning included a general reluctance to move and reduced responsiveness to the presence of other rabbits during the exercise periods. Inappetance and inactivity became generally more pronounced in the one or two days preceding death. Evidence of physical injury associated with anticoagulation was observed in 10 of the 27 rabbits that ingested lethal doses, including lameness favouring one limb, uncoagulated bleeding from minor wounds or visible subcutaneous bruising. In particular, severe haematoma in or around one eye triggered alternative endpoint euthanasia in six rabbits (Table 3), and one of these had a freely bleeding wound in the anogenital area. Noting that the estimated times to death for these six euthanased rabbits were conservative, the overall average time to death from first signs of poisoning was 2.26 days (±0.33) and there was no significant difference in time to death (F_2,24_ = 0.0336, *p* = 0.97) or rabbit survival (χ^22^ = 0.048, *p* = 0.99) between the three trials.

Laboratory rabbits that had died of poisoning or were euthanased at an alternative endpoint had (at least) one large internal haemorrhage which was ascribed as causing death. The haemorrhage sites from the most to least common were; in cardiac and/or thoracic cavity, in hind or forelimbs extending down into the paws, haemorrhage around the gut including the outer stomach wall, intestines and mesenteric membranes, and in the muscle of the hind quarters, shoulder or neck. Most bleeding in limbs occurred in the outer muscle layers and was usually extensive throughout an affected limb, but bleeding into bone joints was not observed. Necropsy also showed no consistent patterns of haemorrhage in rabbits related to the magnitude of the lethal dose ingested, and death in poisoned rabbits was considered due primarily to hypovolaemic shock with contributing effects of anaemia.

Of the nine rabbits that ate sublethal amounts of bait (Table 3), signs of poisoning were observed in only one. This rabbit displayed anaemia, inactivity, lameness and visible bruising of one testicle from 11–15 days after first exposure to bait, but recovered and was apparently healthy at the end of the 21 day monitoring period. Necropsy found the remains of bruising around the right testicle along with bruising between the skin and upper muscle layer of the right hind leg, which extended to the base of the tail. The remains of some small point-haemorrhages were also visible in one lung. We surmise that this rabbit came very close to a lethal extent of anticoagulation, noting that some rabbits survived pindone exposures that were lethal to others (Table 3). The lack of statistically significant differences in amount of pindone ingested versus times to first visible signs of poisoning, or times to death, suggests that there is a variable toxic threshold (perhaps dependent on some physiological status, activity or minor injury that may predicate haemorrhage) and no apparent dose-response effect for pindone poisoning in rabbits *i.e.*, the magnitude of a lethal exposure does not significantly influence the extent or duration of the effects of poisoning.

### 3.2. Field-Collected Rabbits

Of twelve rabbit carcasses collected following a pindone baiting operation at Lake Tekapo, three could not be fully necropsied because they had been partially scavenged. In the nine Tekapo rabbits necropsied there was no visible external bleeding, their stomachs were at least half full and there was no haemorrhaging in the cardiac area or limbs. In seven of these rabbits extensive haemorrhage was concentrated around the gut and mesentery. One rabbit also had a pale mottled liver, suggesting disease may have contributed to mortality. Necropsy of nine rabbits collected after a pindone baiting operation near Wanaka revealed some visible external bruising and more generalised haemorrhage in the abdominal cavity and the musculature of limbs compared to the rabbits from Tekapo. The most likely cause of death for four of the rabbits from Wanaka was abdominal haemorrhage. Two other individuals had haemorrhaged in the hind quarters, and no major identifiable haemorrhage was observed in the remaining three rabbits (although they did have widely distributed free blood in the abdominal cavity). Compared to the necropsy observations of laboratory rabbits, field-collected rabbits showed much more diffuse haemorrhage that was less easily defined as originating from particular tissues or locations and a greater incidence of free, uncoagulated blood pooled in the abdominal cavity. This was considered at least partially due to the difference in the time between death and necropsy in laboratory versus field-collected rabbits, including effects of frozen storage e.g., haemolysis, in the field-collected rabbit carcasses and subsequent defrosting for necropsy.

### 3.3. Nature, Extent and Duration of the Effects of pindone Poisoning in Rabbits

Previous studies have described time to death from pindone poisoning in rabbits. Eason and Jolly [22] orally dosed six laboratory rabbits with 25 mg/kg pindone; three of these died on day 5 or 6 after dosing and the others were subsequently euthanased, preventing a more precise upper range estimate of time to death. Oliver and Wheeler [23] reported times to death of 5–20 days in laboratory rabbits poisoned by pindone and a field study of radio-tracked rabbits after a pindone baiting operation estimated times to death of 6 to16 days [24]; both estimates are consistent with the 6 to 17 days to death found in our study. Similar to our necropsy findings, previous studies also reported observations of extensive internal haemorrhage in poisoned rabbits in various locations; in the abdomen and thorax [22] in the abdominal cavity, in muscle around the rib cage and in the submandibular region, and numerous smaller subcutaneous over the body with small focal haemorrhages in most internal organs [23]. While time to death and necropsy observations provide general indications of the nature and potential maximum duration of effects, our data more fully describe the onset and progression of pindone poisoning in rabbits. After toxic bait was first eaten there was a ‘lag’ period of 6 to 11 days before signs of behavioural change or functional impairment were evident (to a human observer), suggesting that welfare impacts during this period were none to mild and at the lower end of the impact scale. Once signs of pindone poisoning were evident, in all but one rabbit these progressed through to mortality and indicated an increasing level of functional impairment resulting from anticoagulation. The period over which welfare impacts were likely to be moderate to severe (*i.e.*, from first signs of poisoning to death) following lethal pindone exposure ranged from 1.43–4.33 days (Table 3) noting there was no evidence of reduced consciousness before death. Sublethal exposure to pindone may also result in less prolonged or severe welfare impacts; in the trials one rabbit showed signs of poisoning over about 4 days before recovering. 

Functional impairment associated with pindone poisoning in rabbits is most likely to be related to the haemorrhagic outcomes of anticoagulation, which occurred in variable locations and to varying extents. Case studies in humans indicate that anticoagulant-induced haemorrhages in some sites are painful [8]: approximately 42% of the laboratory rabbits in our trial displayed symptoms or behaviour indicative of pain during the later progression of pindone poisoning e.g., observations of swollen joints, reluctance to move or outright lameness, that were linked to later necropsy observations of periarticular haemorrhage around leg joint(s) and musculature. Other observations of functional impairment included inappetance and lethargy, we attribute to blood loss (anaemia) through extensive internal haemorrhage. We could not determine through our observation of live animals whether breathlessness was experienced in the latter stages of poisoning, but this seems likely to have occurred in at least some rabbits given the common post-mortem observation of extensive haemorrhage into the thoracic or cardiac cavity. In free-ranging wild rabbits, reduced mobility through haemorrhage and/or lethargy also has potential for impacts in other welfare domains, such as environmental exposure or alteration in social behaviour e.g., reduced ability to avoid aggressive conspecifics or predators. 

### 3.4. Relative Welfare Impacts of VTAs and Other Lethal Methods for Rabbit Control

**Figure 1:**
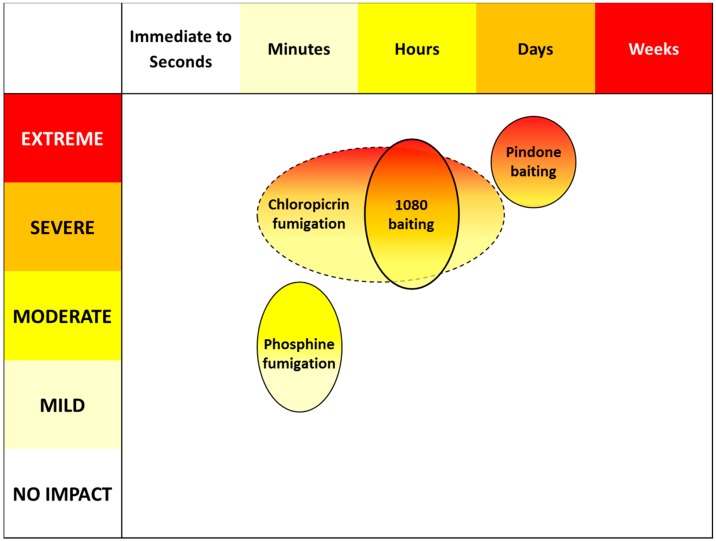
Relative animal welfare impacts of vertebrate toxic agents used for the control of European rabbits (*Oryctolagus cuniculus*), based on rankings from [14].

We present new data on behavioural responses of rabbits to pindone poisoning, in particular around the duration of welfare impacts and negative affective experiences. Application of this data to the welfare impact assessment framework substantially reduces the uncertainty in ranking of pindone as causing severe to extreme welfare compromise in rabbits primarily through functional impairments (Domain 3) that produce negative affective states (Domain 5), over a maximum duration of 7 days (Table 3). ‘Time to death’ was not an accurate metric of the duration of welfare impacts (Table 3): it took at least 6 days after ingestion of lethal quantities of pindone bait for rabbits to display observable signs of welfare compromise. The VTAs currently permitted for operational use for rabbit management in Australia and New Zealand include pindone and sodium fluoroacetate (1080) delivered as toxic baits and the burrow fumigants chloropicrin and phosphine. Based on rankings produced by [14], pindone has the highest welfare impact of these VTAs (Figure 1). Consistent with this ranking, other authors [25] have described pindone as inhumane in comparison to 1080 and some other rabbit control methods. We suggest greater utility of the term ‘animal welfare impact’ rather than ‘humaneness’ in this context because truly humane methods of pest animal management (*i.e.*, lethal or non-lethal methods without welfare impacts) are considered rare (e.g., [14]).Relative rankings of impacts better accommodate comparisons between disparate control methods on a broader scope than the either/or binary implied by the terms ‘humane’ and ‘inhumane’. 

When selecting control methods for unwanted animals there is an expectation that the degree and extent of animal welfare compromise will be minimised where possible [26]. However practitioners must balance welfare against other criteria such as cost-efficacy, human and environmental safety and target-specificity, and in some cases this can mean that a control method with relatively high welfare impacts is the most appropriate to use. A model Code of Practice for humane rabbit management [25] provides an inclusive overview of these aspects for currently available rabbit control methods in Australia. For example while ground-based shooting of rabbits can have relatively low welfare impacts (e.g., [26]), it is labour intensive and inefficient for managing large rabbit populations and not suitable for use near human habitation. Conversely, pindone baiting as a rabbit control method is an option for use in areas close to human habitation, largely because it presents a reduced risk to humans and their domestic animals in comparison to other methods [27], and is less labour-intensive and costly than trapping. There is a clear need for rabbit control methods that are suitable for use in such situations, which have a reduced welfare impact relative to pindone. In this context, the successful development of improved applications of RHDV for rabbit biocontrol [28] may facilitate reductions in pindone use.

## 4. Conclusions

Within an ethically defensible approach to pest animal management, improved information about the relative welfare impacts of different control methods is important to enable both on-ground managers and regulatory agencies to optimise choices in everyday practice and policy making. Here we have added to the information base regarding the animal welfare impacts of one method for rabbit management (pindone baiting), which potentially affects many thousands of rabbits annually. The relatively high animal welfare impacts of pindone poisoning, in comparison to at least other vertebrate toxic agents, suggest that the use of this control method should be limited to situations where there are no other acceptable or appropriate alternatives for mitigating the unwanted impacts of rabbits on natural environments and production values.

## Figures and Tables

**Table 1:** Amounts of pindone bait offered over time to laboratory rabbits in three trials.

Trial	Treatment
1 (*n* = 12)	Estimated effective lethal quantity of bait (4 grams bait/ kg rabbit bodyweight), spread over seven days
2 (*n* = 12)	c. 200 g of pindone pellet bait (approximating 25 mg/kg pindone exposure for each rabbit) for 24 h while normal diet was removed
3 (*n* = 12)	c. 100 g of pindone bait daily for 7 days, so that bait was constantly available in the presence of normal diet

**Table 2:** Scoring system used to monitor laboratory rabbits following ingestion of pindone bait.

Score	Expected Signs of Pindone Poisoning
A	Lethargic, inappetance, depressed carriage, poor grooming
B	Pale mucous membranes, laboured breathing, reluctance or difficulty moving, black/tarry/runny droppings
C	Haemorrhaging obvious e.g., bruising visible, blood from orifice(s); swollen joints or lameness
D	Prostration, recumbency or loss of full mobility, irregular heart beat or respiration

**Table 3:** Numbers of laboratory rabbits that ingested lethal and sublethal amounts of pindone bait, and the average days to the first observed signs of poisoning and to death in rabbits that died of poisoning or were euthanased at an alternative endpoint.

Trial	Died of Poisoning Alternative Endpoint Survived	Mean (Range) Total Pindone Ingested (mg/kg)	Average Days to First Signs of Poisoning (Range)	Average Days to Death (Range)	Average Days from First Signs to Death (Range)
**Trial 1**	8/12	18.11 (13.96–23.41)	8.13 (6–9)	10.63 (8–14)	2.50 (1–5)
	1/12	24.29 (-)	10 (-)	11 (-)	1.0 (-)
	1/12	13.20 (1.69–21.59)	none observed	na	na
**Trial 2**	6/12	26.35 (22.5–30.03)	6.83 (6–8)	9.17 (7–11)	2.60 (1–4)
	2/12	19.11 (16.73, 21.60)	8.50 (6–11)	10.0 (8–12)	1.50 (1–2)
	4/12	21.54 (8.23–30.08)	11 *, none observed	na	na
**Trial 3**	7/12	41.8 (23.8–61.7)	10.43 (8–13)	11.86 (10–13)	1.43 (<0.5–2)
	3/12	40.0 (24.4–60.8)	8 (6–10)	12.33 (8–17)	4.33 (<0.5–7)
	2/12	0	none observed	na	na

* Signs of poisoning observed in one of four sublethally exposed rabbit, but none observed in the other three rabbits.

## References

[1] Fraser D., MacRae A.M. (2011). Four types of activities that affect animals: Implications for animal welfare science and animal ethics philosophy. Anim. Welf..

[2] Meerburg B.G., Brom F.W.A., Kijlstra A. (2008). The ethics of rodent control. Pest Manag. Sci..

[3] Littin K.E., Fisher P., Beausoleil N., Sharp T. (2014). Welfare aspects of vertebrate pest control and culling: Ranking vertebrate control techniques for humaneness. OIE Sci. Tech. Rev..

[4] Thompson H.V., King C.M. (1994). The European Rabbit: The History and Biology of a Successful Colonizer.

[5] Cooke B.D. (2014). Australia’s War against Rabbits: The Story of Rabbit Haemorrhagic Disease.

[6] Williams K., Parer I., Coman B., Burley J., Braysher M. (1995). Managing Vertebrate Pests: Rabbits.

[7] Kirkwood J.K., Sainsbury A.W., Bennett P.M. (1994). The welfare of free-living wild animals: Methods of assessment. Anim. Welf..

[8] Broom D.M., Cowan D.P., Feare C.J. (1999). The welfare of vertebrate pests in relation to their management. Advances in Vertebrate Pest Management.

[9] Littin K.E., O’Connor C.E. (2002). Guidelines for Assessing the Welfare Impacts of Vertebrate Poisons.

[10] Littin K.E., O’Connor C.E., Gregory N.G., Mellor D.J., Eason C.T. (2002). Behaviour, coagulopathy and pathology of brushtail possums (*Trichosurus vulpecula*) poisoned with brodifacoum. Wildl. Res..

[11] Littin K.E., Gregory N.G., Airey A.T., Eason C.T., Mellor D.J. (2009). Behaviour and time to unconsciousness of brushtail possums (*Trichosurus vulpecula*) after a lethal or sublethal dose of 1080. Wildl. Res..

[12] Sharp T., Saunders G. (2011). A Model for Assessing the Relative Humaneness of Pest Animal Control Methods.

[13] Beausoleil N.J., Mellor D.J. (2014). Advantages and limitations of the Five Domains model for assessing welfare impacts associated with vertebrate pest control. N. Z. Vet. J..

[14] Fisher P., Beausoleil N.J., Warburton B., Mellor D.J., Booth L. (2010). How Humane Are Our Pest Control Tools?.

[15] Watt B.E., Proudfoot A.T., Bradberry S.M., Vale J.A. (2005). Anticoagulant rodenticides. Toxicol. Rev..

[16] Paparella M. (2006). Rodenticides: An animal welfare paradox?. Altex.

[17] Beder S., Gosden R. (2010). Pindone rabbit-baiting: Cruel and careless?. Chain Reaction.

[18] Fisher P., Brown S., Arrow J. (2015). Pindone residues in rabbit tissues: Implications for secondary hazard and risk to non-target wildlife. Wildl. Res..

[19] Warburton B., Norton B.G. (2009). Towards a knowledge-based ethic for lethal control of nuisance wildlife. J. Wildl. Manag..

[20] Fisher P. (2005). Review of House Mouse (Mus musculus) Susceptibility to Anticoagulant Poisons.

[21] Littin K.E., O’Connor C., Eason C., Zydenbos S. Comparative Effects of Brodifacoum on Rats and Possums. Proceedings of the New Zealand Plant Protection Conference.

[22] Eason C.T., Jolly S.E. (1993). Anticoagulant effects of pindone in the rabbit and Australian brushtailed possum. Wildl. Res..

[23] Oliver A.J., Wheeler S.H. (1978). The toxicity of the anticoagulant pindone to the European rabbit *Oryctolagus cuniculus* and the sheep Ovis aries. Aust. Wildl. Res..

[24] Robinson M.H., Wheeler S.H. (1983). A radio tracking study of four poisoning techniques for control of the European rabbit, *Oryctolagus cuniculus* (L.). Aust. Wildl. Res..

[25] Sharp T., Saunders G. Model Code of Practice: Humane Control of Rabbits. http://www.pestsmart.org.au/wp-content/uploads/2012/09/rabbitCOP2012.pdf.

[26] Hampton J., Forsyth D., Mackenzie D., Stuart I. (2015). A simple quantitative method for assessing animal welfare outcomes in terrestrial wildlife shooting: The European rabbit as a case study. Anim. Welf..

[27] Fisher P. (2013). Non-Target Risks of Using 1080 and Pindone for Rabbit Control.

[28] Invasive Animals CRC (2014). Import and Evaluate New Rabbit Haemorrhagic Disease Virus (RHDV) Variants to Strengthen Rabbit Biocontrol.

